# Psi promotes *Drosophila* wing growth via direct transcriptional activation of cell cycle targets and repression of growth inhibitors

**DOI:** 10.1242/dev.201563

**Published:** 2023-01-24

**Authors:** Olga Zaytseva, Naomi C. Mitchell, Damien Muckle, Caroline Delandre, Zuqin Nie, Janis K. Werner, John T. Lis, Eduardo Eyras, Ross D. Hannan, David L. Levens, Owen J. Marshall, Leonie M. Quinn

**Affiliations:** ^1^ John Curtin School of Medical Research, The Australian National University, Canberra, ACT 2600, Australia; ^2^ Menzies Institute for Medical Research, University of Tasmania, Hobart, Tasmania 7000, Australia; ^3^ National Cancer Institute, NIH, Bethesda, MD 20892, USA; ^4^ Cornell University, Ithaca, NY 14850, USA

**Keywords:** FUBP1, Psi, Drosophila, Transcription, Myc, Cell cycle

## Abstract

The first characterised FUSE Binding Protein family member, FUBP1, binds single-stranded DNA to activate *MYC* transcription. Psi, the sole FUBP protein in *Drosophila*, binds RNA to regulate P-element and mRNA splicing. Our previous work revealed pro-growth functions for Psi, which depend, in part, on transcriptional activation of Myc. Genome-wide functions for FUBP family proteins in transcriptional control remain obscure. Here, through the first genome-wide binding and expression profiles obtained for a FUBP family protein, we demonstrate that, in addition to being required to activate *Myc* to promote cell growth, Psi also directly binds and activates *stg* to couple growth and cell division*.* Thus, Psi knockdown results in reduced cell division in the wing imaginal disc. In addition to activating these pro-proliferative targets, Psi directly represses transcription of the growth inhibitor *tolkin* (*tok*, a metallopeptidase implicated in TGFβ signalling). We further demonstrate *tok* overexpression inhibits proliferation, while *tok* loss of function increases mitosis alone and suppresses impaired cell division caused by *Psi* knockdown. Thus, Psi orchestrates growth through concurrent transcriptional activation of the pro-proliferative genes *Myc* and *stg*, in combination with repression of the growth inhibitor *tok*.

## Teaser

**Summary:** Psi orchestrates wing growth in *Drosophila* via direct transcriptional activation of pro-proliferative genes in combination with repression of growth inhibitory pathways.

## INTRODUCTION

Human far upstream binding protein 1 (FUBP1) was isolated over a quarter of a century ago for its capacity to bind the far upstream sequence element (FUSE) 1.7 kb upstream of the transcriptional start site for the *MYC* oncogene (Duncan et al., 1994). FUBP1 preferentially binds the single-stranded FUSE, and the FUBP1-FUSE interaction remodels the *MYC* promoter to regulate RNA Polymerase II (Pol II) promoter escape and, thus, fine-tunes transcription (He et al., 2000; Liu et al., 2006). FUBP proteins are conserved throughout evolution, with orthologues in multiple metazoan species. Human and mouse FUBP1 proteins show 98% homology at the amino acid level, with particularly strong conservation within the four KH domains that enable interaction with single-stranded DNA/RNA. As in mammals, there are three members of the FUBP family in other vertebrates, including zebrafish, chicken and *Xenopus* (Li et al., 2006). Functional characterisation of the non-mammalian FUBP proteins has been limited, although mRNA binding has been demonstrated for the FUBP2 orthologue in *Xenopus* and chicken, suggesting RNA-related functions are conserved (Gu et al., 2002; Kroll et al., 2002). The *C. elegans* proteome also contains proteins with sequence and structural similarity to FUBP, indicating an evolutionary origin in simpler invertebrate systems (Davis-Smyth et al., 1996). On the other hand, orthologs are not apparent in yeast, suggesting the FUBP family may have evolved for specific functions in multicellular animals. The three FUBP proteins are conserved as a single orthologue, called Psi (P-element somatic inhibitor) in *Drosophila*, that is structurally similar to the mammalian FUBP1 protein, possessing four central KH domains (Zaytseva and Quinn, 2018).

Although FUBP1 has been implicated in transcriptional control of a handful of other cell cycle control and survival genes (Debaize et al., 2018; Rabenhorst et al., 2009), genome-wide functions for FUBP family proteins, and implications for animal development, have remained unclear. In *Drosophila*, the three mammalian FUBP proteins are represented by one ortholog, Psi, that binds RNA via KH motifs to control RNA splicing (Labourier et al., 2002; Wang et al., 2016), and also promotes cell and tissue growth during development through activation of *Myc* expression (Guo et al., 2016). However, our previous observation that *Psi* knockdown (KD) impairs wing growth more strongly than *Myc* KD (Guo et al., 2016), suggests that the influence of Psi extends to additional growth regulators. Moreover, through interaction with the Mediator (MED) complex (Guo et al., 2016), Psi has capacity to sense and respond to upstream signals in order to regulate downstream transcriptional targets required for cell cycle patterning during development.

We therefore sought to identify direct targets of Psi-dependent growth control using genome-wide binding and expression profiling. Targeted DamID (TaDa) (Marshall et al., 2016; Southall et al., 2013), to profile genome-wide Psi enrichment specifically in the wing imaginal disc, revealed that in addition to directly binding cell cycle-promoting genes, including *Myc* and *stg*, Psi was enriched on genes implicated in developmental signalling. Moreover, RNA sequencing (RNA-seq) detected both up- and downregulation of direct targets in Psi-depleted wings, implying Psi not only behaves as a transcriptional activator (as in the case of *Myc*) but can also function as a repressor. Here, we identify *tolkin* (a metallopeptidase implicated in TGFβ signalling) as a key target of Psi repression and further demonstrate that Tok functions to inhibit cell division; *tok* overexpression reduces proliferation and its loss of function is sufficient to increase mitosis alone and suppress the *Psi* KD phenotype.

Although Psi has been implicated in transcription and splicing, which are often tightly coupled, such that defective transcription can indirectly alter splicing, we observe limited overlap between differentially expressed and spliced genes in Psi-depleted wings. The transcriptional and splicing functions of Psi are, therefore, largely independent. Furthermore, splicing changes were not observed for direct targets necessary for Psi-dependent wing growth (i.e. *Myc*, *stg* and *tok*), inferring Psi promotes tissue growth through transcriptional mechanisms rather than splicing functions. Collectively, our data demonstrate that Psi promotes tissue growth through combined direct activation of cell cycle target genes and repression of developmental growth inhibitors.

## RESULTS

### Psi is required for cell cycle progression

**Fig. 1.**
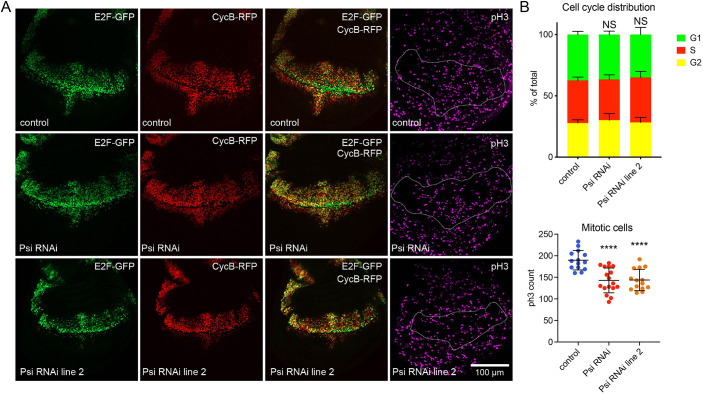
**Psi depletion reduces proliferation.** (A) Third instar larval wing discs with *ser*-GAL4 expression of *UAS*-*FUCCI* with two alternate *Psi* RNAi lines, or control, stained using anti-pH3 antibody. (B) Quantification of the proportion of cells undergoing each cell cycle stage (*n*>10), and the total number of mitotic cells. NS, no significance; ****P<0.0001 when compared with control (*t*-test). Each data point represents a single wing disc. Data are mean±s.d.

We have previously demonstrated Psi KD in the dorsal wing compartment impairs growth of the adult wing (Guo et al., 2016). To determine the cellular basis of reduced wing size, we analysed cell cycle progression, cell growth and cell death following *Psi* knockdown (KD) in larval wings using two independent *Psi* RNAi lines, previously shown to deplete Psi mRNA and protein efficiently (Guo et al., 2016). Cell cycle analysis of *Psi* KD wing discs, measured using the fluorescent ubiquitination-based cell cycle indicator (FUCCI) system (Zielke et al., 2014) and anti-phosphohistone 3 (pH3) antibody for mitosis, revealed no change to G1-S phase progression but significantly reduced mitoses (Fig. 1A,B). Based on quantification of activator caspase (Dcp-1), the increased mitosis was not accompanied by induction of apoptosis (Fig. S1A). Psi depletion did not alter cell growth (i.e. accumulation of biomass), measured indirectly by quantifying nucleolar size, which correlates with rDNA transcription and ribosome biogenesis (Mitchell et al., 2015). Thus, Psi depletion does not decrease cell growth, despite the reduction of *Myc* in *Psi* KD wings and the reduced nucleolar size associated with Myc depletion (Zaytseva et al., 2020). This, together with the reduced mitosis, suggests Psi controls tissue growth via targets in addition to *Myc*.

### Psi associates with euchromatin

Polytene chromosomes in the *Drosophila* salivary gland arise from numerous rounds of endoreplication in the absence of mitosis (Edgar and Orr-Weaver, 2001), thus providing a system for monitoring the extent of Psi binding genome wide. We observed multiple bands of Psi-antibody staining on polytene chromosomes. Relatively strong Psi banding overlapped less intense DAPI staining (Fig. S2A), i.e. regions of open chromatin/active transcription (Lis, 2007), suggesting Psi predominately binds euchromatic targets. To identify Psi targets mediating wing growth (i.e. in addition to Myc) we used Targeted DamID (TaDa), which was developed in *Drosophila* for cell lineage- and tissue-specific genome-wide binding studies (Marshall et al., 2016; Southall et al., 2013). Direct Psi targets differentially expressed (DE) in *Psi*-KD wing discs, compared with control, were determined via intersection of significant Psi-bound targets from DamID with significantly DE genes identified with RNA-seq (Fig. S2B). As an additional measure of transcriptional activity of Psi target genes, global RNA polymerase (Pol) binding was mapped using RNA Pol TaDa, which detects binding of Polr2F (orthologous to human RPABC2), a subunit common to all three RNA polymerases (Filion et al., 2010). Before bioinformatic analysis, TaDa sample quality and consistency between the three biological replicates was confirmed for Psi and Pol using pairwise Spearman correlation (Fig. S3). This analysis also revealed strong correlation between Psi and RNA Polymerase-binding profiles (ranging from 0.53 to 0.8), further suggesting Psi binding overlaps transcriptionally active regions of the genome.

**Fig. 2.**
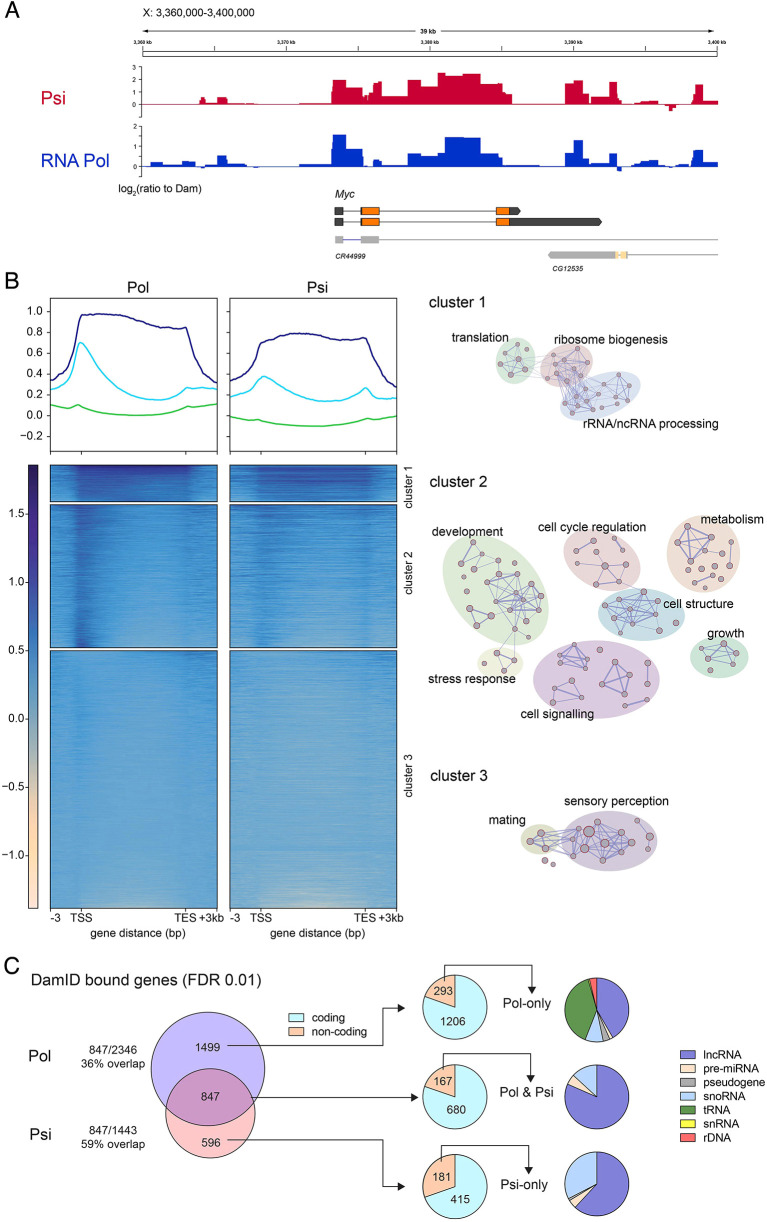
**Psi binds multiple genomic regions, including *Myc*.** (A) Psi- and RNA Pol II-binding profiles across the *Myc* gene in larval wing discs (*sd*-GAL4 driver used for targeted Psi-DamID and RNA Pol-DamID), shown as log_2_ of the ratio to Dam-only control. (B) Average Pol and Psi binding across genic regions and heatmap of DamID signal, clustered by k-means into three clusters using Pol signal. Ontology networks containing common genes and functions are highlighted and labelled manually with general terms. (C) Intersection of Pol and Psi genes with significant enrichment (FDR 1%). Proportions of coding and non-coding genes are shown for each subset, and non-coding genes are further classified by type.

Reassuringly, significant enrichment (normalised log_2_ ratio between Dam-fusion Psi profile and Dam alone) was observed for *Myc,* the prototypical transcriptional target of Psi in the wing,(Fig. 2A). Of note, Psi was not only detected in proximity to the *Myc* transcription start site, consistent with roles in initiation, but was also bound throughout the gene body. These observations suggest transcriptional elongation functions for Psi downstream of pre-initiation complex assembly and/or association with co-transcriptional splicing machinery. Consistent with transcriptional elongation functions, we have previously demonstrated that Psi is required for enrichment of phosphorylated initiating (Ser5) and elongating (Ser2) Pol II on *Myc* (Guo et al., 2016).

In accordance with Psi banding to euchromatic polytene bands (Fig. S2A), and strong correlation between Psi and Pol binding profiles by Spearman correlation (Fig. S3), broad overlap between Psi and Pol enrichment by heatmap clustering further suggests that Psi binding correlates with active transcription (Fig. 2B). Using k-means, three major gene clusters of Psi and RNA Pol binding were identified (Fig. 2B). Cluster 1 displayed high levels of both RNA Pol and Psi DamID signal throughout the body of the gene, and ontology analysis identified enrichment for ribosomal assembly and translation factors (Fig. 2B and Fig. S4A), processes of high demand in third instar wing discs undergoing proliferative growth. A predominant development, signalling and cell cycle signature was observed for cluster 2 genes, which were strongly bound by both Pol and Psi at transcription start sites relative to the gene body (Fig. 2B and Fig. S4B). Cluster 3 genes, bound at low levels by both Psi and Pol, were enriched for neurosensory perception and mating, processes that are expected to be transcriptionally repressed in larval wing discs (Fig. 2B and Fig. S4C).

Genome-wide, closer analysis of statistically significant peaks (Table S1) revealed that Psi bound 1443 genes (9.2% of the 15,682 genes annotated in Ensembl 73), with 847 (59%) of Psi targets also enriched for RNA Pol (Fig. 2C). Non-coding RNA comprised 12.4% RNA Pol-binding targets, and included Pol II-bound long non-coding (lncRNA), small nuclear (snRNA), small nucleolar (snoRNA) and pre-miRNAs. In addition to coding and non-coding genes regulated by Pol II, because the common RNA Pol subunit was used for TaDa, significant Pol I enrichment was detected on ribosomal DNA (rDNA) and Pol III enrichment on transfer RNA (tRNA) loci (Fig. 2C). Although 24% of the targets of Psi were non-coding, these were confined to targets of Pol II transcriptional control (lncRNAs, snoRNAs and miRNAs) i.e. Psi binding was not detected on rDNA or tRNA loci (Fig. 2C). Together with the co-enrichment for Psi and Pol on ribosomal assembly and translation factor genes (Fig. 2B and Fig. S4A), these observations suggest Psi-dependent cellular growth is mediated by Pol II transcription rather than through direct effects on rDNA or tRNA transcription.

### Psi independently regulates gene expression and RNA splicing

**Fig. 3.**
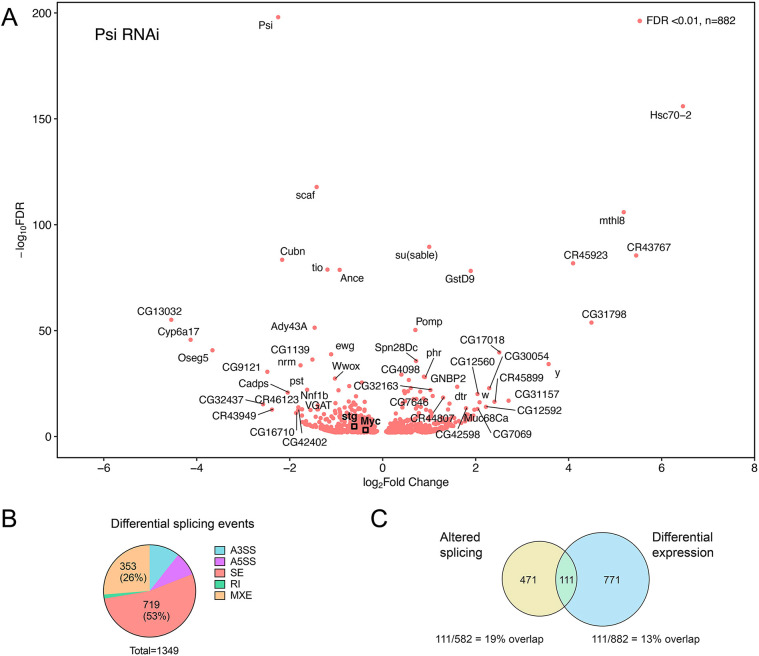
**Significantly altered genes after *Psi* knockdown in wing discs.** (A) Genes with statistically significant altered expression after *Psi* knockdown at FDR<0.01. Top 50 genes with greatest fold change and smallest *P*-value are labelled. *Myc* and *stg* are highlighted with black squares. (B) Proportion of differential splicing events detected by rMATS at FDR<0.01: alternative 3′ splice site (A3′SS), alternative 5′ splice site (A5′SS), skipped exon (SE), retained intron (RI) and mutually exclusive exons (MXE). (C) Intersection of differentially expressed genes with genes exhibiting altered splicing.

RNA-seq for *Psi* KD wing imaginal discs detected 882 differentially expressed (DE) genes compared with control (Fig. 3A, Table S2). As expected, based on our previous studies (Guo et al., 2016), *Myc* mRNA levels were significantly reduced in *Psi* knockdown wing discs (Fig. 3A, log_2_FC=-0.369, adjusted *P*=0.0008). An additional 428 genes were downregulated in *Psi* KD wing discs, while 453 genes were upregulated (Fig. 3A). In addition to binding single-stranded DNA, mammalian FUBP-family proteins can also bind RNA via their KH domains to regulate RNA processing (Gherzi et al., 2014; Miro et al., 2015). As Psi binds RNA via the KH motifs to control RNA splicing (Labourier et al., 2002; Wang et al., 2016), we therefore analysed differential splicing using rMATS (Shen et al., 2014), which identifies mis-spliced events and additionally has the capacity to discover unannotated splice sites. rMATS detected 1349 events at 582 genes with differential splicing for *Psi* KD compared with control (Table S3). Classification into splicing event types by rMATS identified exon skipping (53%) and mutual exon exclusivity (26%) as the most common alterations in *Psi* KD (Fig. 3B). Based on rMATS analysis, *Myc* was not differentially spliced after *Psi* depletion, as downregulation occurred without a relative change in the proportion of reads overlapping introns (Fig. S5A). Thus, Psi predominantly functions to regulate *Myc* at the level of transcription. Together with Psi binding across the *Myc* gene (Fig. 2A), and the requirement for Psi in RNA Pol II loading on *Myc* and maintenance of *Myc* mRNA levels (Guo et al., 2016), our data strongly suggest that Psi regulates *Myc* at the level of transcription.

Ontology analysis of genes differentially spliced in *Psi* knockdown wings, compared with control, detected enrichment for developmental pathways (Fig. S5B). Intersection of expression and splicing data identified 111 genes both differentially expressed and alternately spliced (Fig. 3C). The relatively small overlap (13% alternately spliced and 19% differentially expressed) indicates that most transcriptional and splicing changes occur independently and implying that defective coupling of transcription and splicing, where impaired transcription indirectly alters splicing patterns (Bentley, 2014), is not a major attribute of Psi loss of function. Moreover, differentially spliced and altered genes included splicing regulators SF1, Saf-B and B52 (Brooks et al., 2015; Mayeda et al., 1992; Park et al., 2004), the dysregulation of which may indirectly contribute to splicing defects associated with Psi depletion. In order to elucidate roles of Psi in transcriptional control without confounding effects of differential splicing, subsequent analysis focused on the direct transcriptional targets of Psi that are altered at the level of expression rather than by splicing.

### Direct targets of Psi control development

**Fig. 4.**
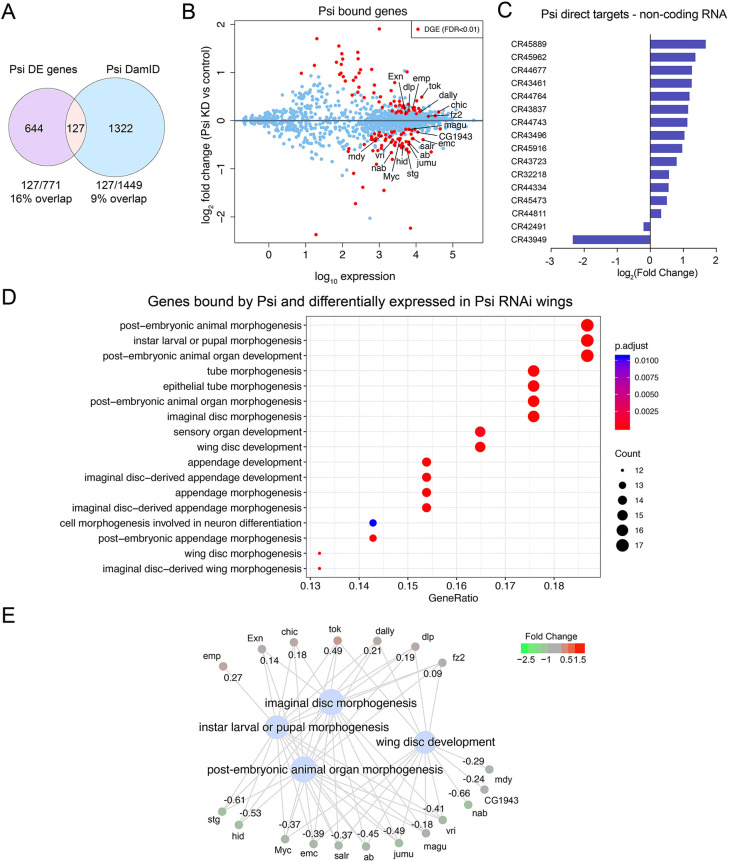
**Psi binds and regulates developmental genes.** (A) Intersection of differentially expressed genes after *Psi* knockdown with genes bound by Psi. (B) MA plot showing only genes bound by Psi (blue) while statistically significant DGE events at FDR<0.01 are shown in red. (C) Fold change and expression of ncRNA Psi targets. (D) Ontology of mutually inclusive genes from the intersection in A. (E) Genes regulated by Psi with annotated roles in wing morphogenesis; log_2_(fold change) values are as indicated.

**Fig. 5.**
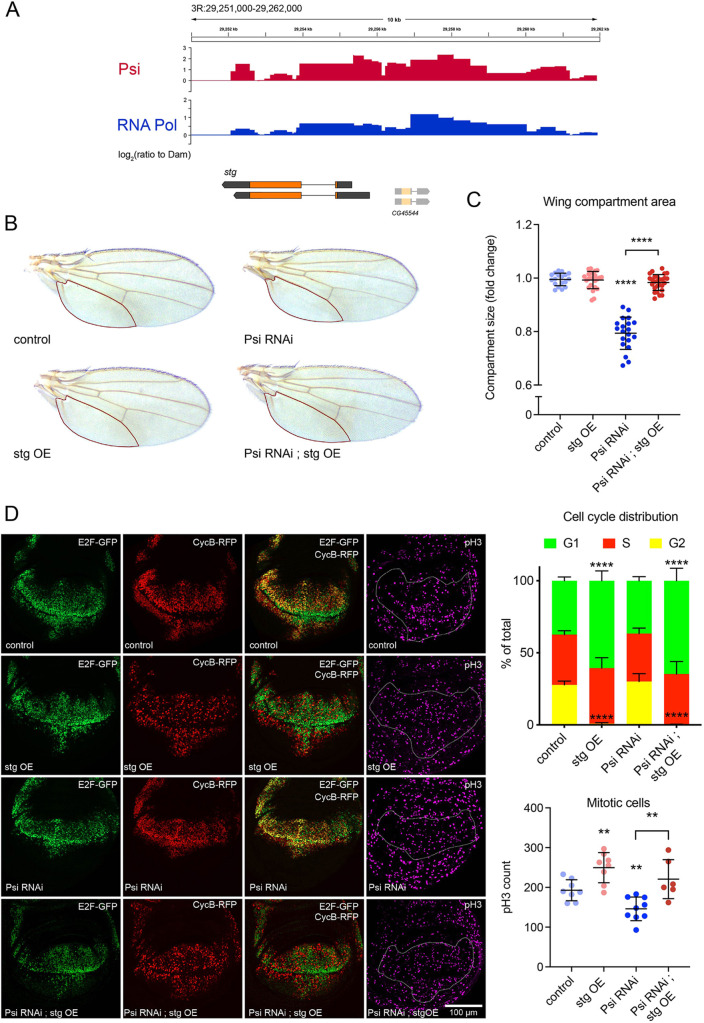
**Stg overexpression rescues impaired growth associated with Psi depletion.** (A) Psi and RNA Pol II binding profiles across the *stg* gene in larval wing discs (log_2_ of the ratio to Dam-only control). (B) Adult wings with *ser*-GAL4 driven knockdown of *Psi* alone or in combination with *stg* overexpression (OE). (C) Quantification of the posterior compartment of the adult wing defined by the L5 vein, marked with a red outline in B. ****P_adj_<0.0001 (corrected for multiple testing using the Benjamini-Hochberg FDR method). (D) Third instar larval wing discs with *ser*-GAL4 expression of *UAS*-*FUCCI* with genotypes as marked, stained using anti-pH3 antibody. Quantification of the proportion of cells undergoing each cell cycle stage (*n*>6) and the total number of mitotic cells. ****P<0.0001 compared with control, **P<0.01 (*t*-test). Each data point represents a single adult wing or wing disc. Data are mean±s.d.

The intersection of the *Psi* KD transcriptome and DNA-binding profiles, used to identify the direct and differentially expressed targets of Psi, identified 127 genes shared between the two gene sets (Fig. 4A, Table S4). Genome-wide, 16% of DE genes in *Psi* KD wing discs were directly bound by Psi, while 84% of the DE genes were likely controlled indirectly. Of note, given the requirement for mitosis (Fig. 1), Psi was enriched on the essential mitotic phosphatase *Cdc25* (also known as *stg*), and required for maintaining endogenous levels of *stg* expression (Figs 5A and 3A)*.* As observed for *Myc*, splicing of *stg* was not altered by *Psi* knockdown (Fig. S5C), indicating Psi regulates *stg* at the transcriptional level. Moreover, *stg* overexpression restored tissue size in *Psi* KD wings (Fig. 5B,C; Fig. S7A), suggesting decreased *stg* expression is required for impaired growth in Psi-depleted wings. *Stg* overexpression alone, or in the background of Psi depletion, dramatically reduced the proportion of G2 cells and increased mitoses (Fig. 5D). Thus, Psi likely drives wing growth by upregulating *Myc*, and couples S-phase progression with entry into mitosis via upregulation of *stg*.

However, Psi did not invariably activate Pol II-dependent transcription, as observed for *Myc* and *stg*, rather, direct Psi targets were equally up- (64) and downregulated (63) in *Psi* KD wings (Fig. 4B). Among the direct targets of Psi were 16 long non-coding RNAs, most of which (14/16) were upregulated, consistent with repression by Psi (Fig. 4C). Although the function of these lncRNAs is unknown, CR44811 has been linked with developmental growth control: being activated by the Yki transcriptional co-activator downstream of Hippo in wing discs (Zhang et al., 2017).

Ontology analysis of direct and differentially expressed targets revealed that Psi predominantly modulates genes implicated in developmental growth and morphogenesis (Fig. 4D,E). The observation that similar numbers of direct Psi targets were upregulated after *Psi* KD suggested novel transcriptional repressor roles for Psi. Repressed Psi targets, which are crucial for growth inhibition in the wing, would be predicted to rescue the small wing phenotype associated with *Psi* knockdown (Guo et al., 2016). Thus, we sought to identify the directly repressed candidates, previously associated with wing development (including *chic*, *dally*, *dlp, fz2, tok, emp* and *Exn*, Fig. 4E, Fig. S6), that mediate Psi-dependent wing growth. RNAi transgenes without predicted off-targets were validated for *emp*, *fz2*, *chic* and *dally* (Fig. S7B). Impaired wing growth due to *Psi* depletion was not suppressed by co-KD of *emp*, *fz2*, *chic* or *dally* (Fig. S7C), suggesting KD of these targets individually is insufficient to modify Psi-dependent wing growth. However, co-knockdown of *dlp* or *Exn* suppressed the *Psi* KD small wing phenotype (Fig. S8A), indicating these transcriptional targets are crucial mediators of the impaired wing growth associated with *Psi* KD. Importantly, co-expression of a *UAS*-RFP transgene, to control for GAL4 dilution, did not modify the impaired wing growth associated with *Psi* KD (Fig. S7C). Nevertheless, despite the capacity to suppress impaired growth caused by Psi depletion, individual KD of *dlp* (a Dally-like glypican that regulates Wg/Wnt signalling; McGough et al., 2020) significantly reduced wing growth (Fig. S8B), suggesting that *dlp* is required for growth. In contrast, depletion of *Exn* alone was sufficient to promote wing overgrowth (Fig. S8B). A second, independent *Exn* RNAi line similarly suppressed the Psi KD small wing phenotype and increased wing size (Fig. S8C,D).

To determine the cellular basis for altered wing growth, we characterised *Exn* KD larval wing imaginal discs. To control for possible effects of GAL4 dilution, we also verified that co-expression of a *UAS*-RFP transgene did not modify the *Psi* KD larval wing phenotypes (Fig. S8E-G). *Exn* knockdown did not modify mitosis (Fig. S8E) or nucleolar size (Fig. S8F), either alone or in combination with *Psi* KD, but *Exn* KD alone reduced apoptosis (Fig. S8G), suggesting overgrowth may be a consequence of increased cell survival. However, *Exn* co-depletion did not modify apoptosis in *Psi* KD wing discs, indicating suppression of the small wing phenotype by Exn is not likely due to increased survival in the larval stage, although wing size may be restored by increased survival in the pupal stage.

The *tok* RNAi TRIP line (BL66320) efficiently depletes *tok* mRNA without altering the potential off target predicted by dsCheck analysis (*Srrm234*, Fig. S9A,B). *Ser-*GAL4 driven *tok* RNAi KD did, however, result in significantly increased apoptotic cell death and ablation of the dorsal compartment (Fig. S9C). To analyse potential proliferative phenotypes in third instar wings before the induction of cell death, we used GAL80^ts^ for transient induction of *ser-*GAL4-driven *tok* KD. Indeed, fibrillarin staining 24 h after induction of *tok* KD revealed significantly increased nucleolar area, suggesting tok is required for inhibition of cell growth (Fig. S9D). However, the rapid induction of cell death associated with cell overgrowth driven by the *tok* RNAi KD precluded analysis of mitosis in the larval wing disc, while larval lethality prevented analysis of adult wing phenotypes.

**Fig. 6.**
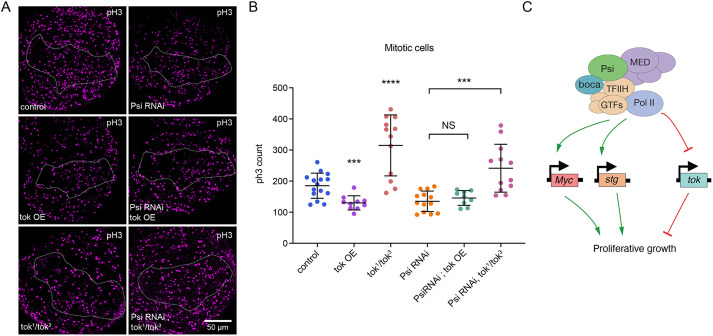
**Repression of *tok* is required for Psi-dependent cell division.** (A) Larval wing discs with *ser*-GAL4-driven *tok* overexpression (OE) and/or *Psi* RNAi, or *tok^1^*/*tok^3^* transheterozygotes with or without *ser*-GAL4 driven *Psi* RNAi KD stained using anti-pH3 to detect mitosis. (B) Quantification of mitotic cells in the *ser*-GAL4 compartment, ****P<0.0001. Each data point represents a single wing disc. Data are mean±s.d. (C) Model for Psi function in regulating proliferative growth during development.

Thus, to further analyse Tok function in the wing, we used previously described *tok^1^* and *tok^3^* loss-of-function mutants (Finelli et al., 1995). To avoid confounding effects of potential off-target mutations in these strains, we analysed wing imaginal discs for *tok^1^*/*tok^3^* transheterozygotes. We observed increased mitoses in *tok^1^*/*tok^3^* larval wings compared with control (Fig. 6A,B). The more severe phenotype for *tok* RNAi compared with *tok^1^*/*tok^3^* transheterozygotes likely reflects the difference between acute depletion of Tok specifically in the dorsal wing compartment with reduced Tok in the whole animal. Specifically, we predict the rapid depletion of Tok results in cellular overgrowth, but this is associated with cellular stress and associated apoptosis, preventing cell cycle progression. Importantly, survival of *tok^1^*/*tok^3^* wing discs enabled modification of the Psi KD mitotic phenotype to be tested without confounding effects of cell death. Not only did *tok^1^*/*tok^3^* transheterozygotes show increased larval wing mitosis, but *tok^1^*/*tok^3^* transheterozygotes restored mitoses in the *Psi* KD dorsal compartment to the control range (Fig. 6A,B). Thus, Tok depletion suppressed impaired cell division associated with Psi KD in the larval wing.

As RNA-seq showed increased *tok* expression in *Psi* KD wings (Fig. 4E), we further tested whether *tok* overexpression (OE) (validated in Fig. S9A) was sufficient to alter wing size. Consistent with growth inhibitory roles, *tok* OE in the dorsal compartment reduced adult wing size alone, but did not further impair growth in *Psi* KD wings (Fig. S9E). *tok* OE did not alter nucleolar size or cell death, either alone or in the background of *Psi* KD (Fig. S9F,G), but significantly decreased wing disc mitosis to levels comparable with *Psi* KD alone (Fig. 6A,B). Thus, the increased *tok* detected in *Psi* KD wings likely contributes to impaired growth, further suggesting that *tok* is a key target of Psi-dependent transcriptional repression in the developing wing. Taken together, our data suggest that Psi promotes cell division and tissue growth through direct activation of the cell cycle target genes *Myc* and *stg*, and through repression of *tok*, which functions as an inhibitor of proliferation during wing development.

## DISCUSSION

Using genome-wide binding and transcriptome profiling, combined with functional studies, we demonstrate that Psi controls proliferative growth during development through transcriptional regulation of multiple targets (Fig. 6F). Myc overexpression in the larval wing disc drives G1- to S-phase progression, but is unable to activate the Cdc25/Stg phosphatase to drive mitotic entry and, thus, results in a G2 delay, which can be overcome by independently increasing Stg (Johnston et al., 1999; Reis and Edgar, 2004). Thus, transcriptional upregulation of *Myc* and *stg* by Psi provides a mechanism to increase both G1-S and G2-M machinery, coupling cell growth and division to enable wing growth.

Although Psi binds and regulates transcription of multiple direct targets, the individual changes to each gene are small, consistent with the function of the homologous mammalian FUBP1 protein in fine-tuning Pol II-dependent transcription (Liu et al., 2006; Zheng et al., 2020). Our data suggest that Psi regulates tissue growth through broad alterations to the transcriptome, i.e. altering numerous target genes, which in combination alter cell growth (e.g. *Myc*) and cell division (*stg* and *tok*). Moreover, indirect targets of Psi have the potential to impact growth. For example, *erect wing genes* (*ewg*) was not directly bound by Psi, but was among the top downregulated genes in *Psi* KD wings (Fig. 3). As a modulator of Hippo and Wg/Wnt pathways (Hsiao et al., 2013; Xin et al., 2011), decreased *ewg* may contribute to impaired wing growth associated with *Psi* depletion.

In addition, we have identified Tok as a novel cell cycle inhibitor and a direct target of Psi repression. The Tok metalloprotease, which is implicated in TGFβ/Dpp pathway signalling, regulates embryonic patterning by cleaving the secreted protein Sog, which in turn activates TGFβ/Dpp (Peluso et al., 2011; Serpe and O'Connor, 2006; Serpe et al., 2005). In the wing, TGFβ/Dpp signalling activates Myc (Doumpas et al., 2013) to drive proliferative growth (Bosch et al., 2017). Our observations that *tok* overexpression inhibits proliferation and impairs wing growth suggest that *tok* is unlikely to do so through activation of Dpp. Consistent with *tok* having additional cleavage targets, recent studies have revealed functions in proteolytic cleavage of the axon-guidance protein Slit (Kellermeyer et al., 2020). Given the significant growth inhibitory functions of *tok* in the wing, unbiased approaches to identify pro-proliferative targets will be of great interest.

Mass spectrometry previously identified the transcriptional Mediator (MED) complex as a major component of the Psi protein interactome (Guo et al., 2016). By sensing cellular signalling inputs, MED modulates context-dependent RNA Pol II transcription and, thus, controls development by integrating diverse signalling networks (Allen and Taatjes, 2015). For example, MED12 and MED13 integrate Wg/Wnt and Notch signals to modulate transcription of downstream target genes and to establish compartment boundaries in the wing (Carrera et al., 2008; Janody and Treisman, 2011; Janody et al., 2003). The endoplasmic reticulum protein Boca, which enables trafficking of the arrow (Arr) receptor that, together with Fz/Fz2, is activated by the Wg/Wnt ligand (Culi and Mann, 2003; Tolwinski et al., 2003), is one of the major protein interacting partners of Psi/MED (Guo et al., 2016). Psi also physically interacts with Dishevelled (Dsh) (Guo et al., 2016), a conserved Wg/Wnt pathway adaptor that, upon activation of Fz/Fz2, sequesters the APC/Axin protein destruction complex to stabilise β-catenin/Armadillo and activate Wg/Wnt transcriptional targets (Bejsovec, 2006). Psi protein is ubiquitously expressed in the wing, i.e. expression does not correlate with expression of major wing patterning pathways (Guo et al., 2016). Psi does, however, undergo phosphorylation (Bodenmiller et al., 2008), suggesting the potential for post-translational regulation of Psi activity by upstream signalling pathways in the wing. Therefore, future studies to determine whether interaction between the Psi/MED transcriptional network and developmental signalling pathways that are crucial for wing growth, including Wg/Wnt, TGFβ/Dpp and Hippo, are warranted.

The first characterised member of the FUSE Binding Protein family, FUBP1, was discovered in human cells. Although FUBP1-like proteins have been annotated in all metazoans, including *C. elegans* (Davis-Smyth et al., 1996), orthologous proteins are not apparent in yeast. In light of our findings, we speculate the FUBP family may therefore have arisen to enable the patterning of cell growth that is essential for the development of multicellular organisms.

## MATERIALS AND METHODS

### Expression constructs

*pTaDaG-Psi* was generated by PCR amplifying the ORF inserts from DRGP plasmid FMO09121 and cloning into the *pTaDaG* vector cut with BglII/XhoI via NEB HiFi Assembly (NEB). PCR primers for NEB HiFi Assembly were designed using PerlPrimer. *pTaDaG-RpII18* was generated via the insertion of a custom gBlock (IDT) containing *cMycNLS-linker-RpII18-RA* ORF into *pTaDaG* cut with BglII/XhoI via NEB HiFi Assembly. Primer and gBlock sequences are provided in Tables S5 and S6.

### Fly stocks

*Drosophila melanogaster* stocks were maintained on a standard molasses and semolina *Drosophila* medium. Genetic crosses were raised at 25°C except when performed in the temperature-sensitive GAL80 background, where they were initially raised at 18°C followed by a shift to 29°C. The *Serrate-*GAL4 (BL6791), *Scalloped-*GAL4 (BL8609), *Tubulin-*GAL4 (BL5138), *Tubulin-*GAL80^ts^ (BL7019), *UAS-chic* RNAi (BL34523), *UAS-dally* RNAi (BL33952), *UAS-dlp* RNAi (BL34091), *UAS-Exn* RNAi (BL33373), *stg OE* (BL4777), *tok OE* (BL20105), *tok* RNAi (BL66320), *tok^1^* (BL4586), *tok^3^* (BL4569) and *UAS-FUCCI* (BL55110) lines were obtained from the Bloomington *Drosophila* Stock Centre. The *UAS-Psi* RNAi (v105135), *UAS-Psi* RNAi line 2 (v28989), *UAS-emp* RNAi (BL53257), *UAS-Exn* RNAi line 2 (v105885) and *UAS-fz2* RNAi (v108998) were obtained from the Vienna *Drosophila* Resource Centre. Targeted DamID lines generated for this study (*TaDaG-psi* and *TaDaG-rpII18*) were generated by BestGene through phiC31-integrase-mediated insertion of the appropriate expression vectors into attP2 on chromosome 3.

### Immunofluorescence, microscopy and image analysis

Crosses were maintained at 25°C. Wandering 3rd instar larvae were dissected and fixed for 20 min in 4% paraformaldehyde (PFA), washed in PBS with 0.1% Tween (PBT), blocked in 5 mg/ml bovine serum albumin (BSA) before incubation overnight at 4°C with primary antibody. Primary antibodies used for immunofluorescence were: mouse anti-fibrillarin (1:1000, Abcam ab4566), rabbit anti-phospho-Histone3 (1:5000, Abcam ab14955) and mouse anti-Dcp1 (1:500, Cell Signaling 9578S). After incubating with appropriate fluorophore-tagged secondary antibodies (Jackson ImmunoResearch: anti-rabbit 488, 1:1000, 711-545-152; anti-mouse 488, 1:1000, 715-545-150; anti-rabbit 680, 1:1000, 711-625-152; and anti-mouse 647, 1:1000, 715-605-151) samples were counterstained with DAPI solution and wing imaginal discs imaged using the Zeiss LSM800 confocal microscope (Zen Blue software). Overlapping 1 μm *z*-sections were collected at 40× magnification. Fluorophores were imaged using band-pass filters to remove cross-detection between channels. Images were processed and prepared using Image J and Adobe Photoshop. Fibrillarin size was quantified in FIJI on confocal *z*-sections of wing columnar epithelial cells, merged to display maximum projections (two or three sections). Thresholding was performed and images were used to measure average Fibrillarin area or cell size in the dorsal compartment marked by *serrate-*GAL4>*UAS*-RFP expression. Fifty to 100 nucleoli were selected using freeform selection tool, and analysed with the ‘Analyse Particles’ tool, with minimum particle size of 0.5 μm^2^ applied to exclude noise and out of focus nucleoli. The output used image metadata to calculate average area in μm^2^ for each wing disc analysed. Total caspase area in the wing pouch was measured in FIJI using the maximum *z*-projection of the entire wing disc. FUCCI/ pH3 analysis was performed in Imaris. Total spot pH3 counts were counted in the Serrate-GAL4 compartment. Spot counts for green and red channels were generated, and the MatLab XTension ‘Colocalize Spots’ used to detect spots that were found in similar 3D coordinates, i.e. colocalised, using a distance threshold of 1 μm. The counts for green-only, red-only and yellow-only cells were expressed as proportion of the total cells counted, corresponding to G1, S and G2 cell cycle phases, respectively.

### Polytene immunostaining

The larvae were heat shocked for 20 min at 36.5°C. Polytene squashes and immunofluorescence labelling was carried out as previously described (Schwartz et al., 2004). The chromosomes were stained with Psi antibody (raised against full-length Psi protein in guinea pigs) at 1:20 and Hoechst 33258 for labelling of DNA.

### DamID sample preparation

Embryos from parental crosses using the *sd*-GAL4 ; *tub*-GAL80^ts^ driver were collected over the course of <4 h lays at 25°C, after which the embryos were placed at the repressive temperature of 18°C for 7 days until the second larval instar stage. The larvae were then shifted to the permissive temperature of 29°C for 24 h. Larval wing discs were collected in ice-cold PBS, genomic DNA was extracted using a Zymo Quick-DNA kit (D4069) after treatment with Proteinase K for 1-3 h at 56°C in the presence of 50 μM EDTA. GATC methyl-specific digest using DpnI was carried out at 37°C overnight, and cleaned up using a Machery-Nagel PCR purification kit (740609.50). Samples were eluted into 30 μl H_2_O and 15 μl was used for subsequent preparation. Adaptors for PCR enrichment of methyl-digested sites were ligated for 2 h at 16°C using T4 DNA ligase. A digest of unmethylated GATC sequences was performed with DpnII at 37°C for 2 h, in order to decrease signal from unlabelled sites. PCR using MyTaq polymerase (Bioline BIO-21,113) was performed with three long extension cycles followed by 17 short extension cycles as described previously (Vogel et al., 2007). The PCR products were cleaned up again with a Machery-Nagel PCR purification kit. PCR adaptors were removed by overnight digest at 37°C with AlwI. Samples were sonicated in 100 μl volumes using a Covaris S2 sonicator at 10% DUTY, 140 W peak incident, 200 cycles per burst and 80 s duration, achieving a 300 bp average fragment size. Sample clean-up and library preparation was carried out using Sera-Mag Speedbeads hydrophobic carboxyl magnetic beads (GE Healthcare, 65152105050250). After bead cleanup, sample concentrations were measured using Qubit DNA HS reagents (Thermo Fisher, Q32854) and <500 ng of DNA for each sample was used to generate the libraries. End repair was performed for 30 min at 30°C with T4DNA Polymerase, Klenow Fragment and T4 polynucleotide kinase. 3′ ends were adenylated using Klenow 3′ to 5′ exo-enzyme for 30 min at 37°C. Unique index adaptors were ligated to each sample using NEB Quick Ligase for 10 min at 30°C. The samples were cleaned up with Sera-Mag beads twice to ensure the removal of sequencing adaptor dimers. DNA fragments were enriched by PCR using NEB Next Ultra II Q5 Master Mix (NEB M0544S), before final clean up using Sera-Mag beads. Successful ligation of adaptors and the absence of adaptor concatemers were verified using an Agilent Bioanalyser, and the final concentration was measured using Qubit. The libraries were pooled to achieve an equimolar concentration of each sample based on average fragment size and concentration, with a final total concentration of 2 nM. The samples were sequenced using a HiSeq2500 Illumina platform in Rapid Run mode with 50 bp single-end reads.

### DamID analysis

The DamID dataset was analysed using a single pipeline workflow (Marshall and Brand, 2015). The damidseq_pipeline script was used to align the reads to the *Drosophila* BDGP6 genome with Bowtie2, to identify GATC sites and to calculate the normalised log_2_ ratio of the Dam-fusion protein profile and Dam alone. Spearman sample correlation and genomic coverage clustered metaplots were generated using the deepTools package (Ramírez et al., 2016), using the output of the damidseq_pipeline bedgraph files converted into bigwig files. To generate representative genome-wide binding profiles, the average enrichment of TaDa samples (3×biological replicates) was calculated at each GATC-flanked genomic fragment. Enrichment profiles in bedgraph format were visualised using the Integrative Genome Viewer (IGV). Significant peaks were detected at 1% FDR using the find_peaks script, peaks2genes script to identify genes within 1 kb of the discovered peaks, and transcriptionally active genes were identified using the polii.gene.call script (Marshall and Brand, 2015).

### RNA-seq

Larval wing discs were collected after 3 days of GAL4-induced knockdown. For each sample, three collections of 20 larval wing discs were pooled (60 wing discs in total). RNA was extracted using the Promega ReliaPrep RNA Tissue miniprep system and eluted in 50 μl nuclease-free water and RNA integrity verified using a Bioanalyser Tapestation. Library preparation was carried out by the ACRF Biomolecular Resource Facility, John Curtin School of Medical Research, Australian National University. RNA was prepared using the standard TruSeq Illumina protocol preserving strandedness information, with Oligo-dT beads used to enrich for mRNA and exclude other RNA. Samples were sequenced using the HiSeq2500 Illumina system, with 100 bp paired-end reads.

### Differential expression analysis

RNAseq sequences were aligned to the *Drosophila melanogaster* genome FlyBase release 6.10 using Tophat2. The gene counts were performed using HTSeq Python package (Anders et al., 2015). Significant differential expression was analysed using DESeq2 R package (Love et al., 2014), with FDR cutoff 1% used to identify statistically significant events.

### Gene ontology analysis

Gene Ontology analysis of Entrez IDs associated with significantly altered genes was performed using the clusterProfiler R package (Yu et al., 2012). The Benjamini-Hochberg multiple testing correction method was used and adjusted *P*-value cutoff of 0.05 was applied. The clusterProfiler filtering function was applied to exclude parent terms, where applicable.

### Differential splicing analysis

Analysis of differential splicing was performed using rMATS 4.0.2 (Shen et al., 2014) on BAM files aligned for differential expression. Junction reads as well as reads covering the exon of interest were used to calculate differences in exon inclusion rates. Adjusted *P*-value cutoff of 0.01 was applied to detect significant splicing changes. The ggsashimi package (Garrido-Martín et al., 2018) was used to generate a sashimi plot of average reads across the *Myc* and *stg* genes.

### qPCR

RNA was isolated from equivalent numbers of wing imaginal discs (10 pairs for each genotype) using the Promega ReliaPrep RNA Tissue miniprep system and eluted in 20 μl nuclease-free water. RNA purity and integrity were assessed using an automated electrophoresis system (2200 TapeStation, Agilent Technologies). 5 μl of RNA was used for each cDNA synthesis (GoScript Reverse Transcription System kit, Promega). qPCR was performed using Fast SYBR Green Master Mix (Applied Biosystems) using the StepOnePlus Real-Time PCR System and Sequence Detection Systems in 96-well plates (Applied Biosystems, 95°C for 2 min, 40 cycles of 95°C for 1 s and 60°C for 20 s). Amplicon specificity was verified by melt curve analysis. Average Ct values for two technical replicates were calculated for each sample. Multiple internal control genes were analysed for stability and target gene expression was normalised to the mean of *cyp1* and *tubulin* alone, which were selected for having high expression and little sample-to-sample variability, as determined by RefFinder. Fold change was determined using the 2-ΔΔCT method.

Primers used were as follows: chic, 5′ TTTACCTTTCCGGCACAGACC 3′ and 5′ TGGAAACGATCACGGCTTGT 3′; dally, 5′CATCATCACACCAGCAGCCT 3′ and 5′ GCCAATTCCAGGACGTGACT 3′; dlp, 5′ TTTCCAAGCGAGAGGAATCG 3′ and 5′ ACCGAAGGGGACTCGCAATA 3′; emp, 5′ GGACCCTACGTTTACAGCGA 3′ and 5′ TGTAGCTCAGCGTGCCATTG 3′; Exn, 5′ CTTAAGGACCAAGCCGGCAA 3′ and 5′ AAGACAACACCAGCTCGACG 3′; fz2, 5′ CGACTGCATGTGACACCAAAG 3′ and 5′ GGGCAATGTCGCCCATGAAA 3′; stg, 5′ TGCTGTGGGAAACTATTGTGGA 3′ and 5′ GCTACTCGAACTGCTGGTGT 3′; tubulin, 5′ TCAGACCTCGAAATCGTAGC 3′ and 5′ AGCCTGACCAACATGGATAGAG 3′; cyp1, 5′ TCGGCAGCGGCATTTCAGAT 3′ and 5′ TGCACGCTGACGAAGCTAGG 3′.

### Adult wing analysis

Adult wings were mounted in paraffin oil. Adult wing size was determined for male wings that were imaged with an Olympus SZ51 binocular microscope, at 4× magnification using an Olympus DP20 camera. Wing size was measured by pixel count for the area posterior to wing vein L5 using FIJI.

### Statistics

All statistical tests that were not part of the RNAseq or DamID analysis were performed with Graphpad Prism 7 using an unpaired two-tailed *t*-test with 95% confidence interval. *P*-values for the adult wing size data were corrected for multiple testing using the Benjamini-Hochberg FDR method. In all figures, the error bars represent s.d. and significance is represented according to the Graphpad classification *P=0.01-0.05, **P=0.001-0.01, ***P=0.0001-0.001 and ****P<0.0001.

## Supplementary Material

10.1242/develop.201563_sup1 Supplementary information
