# Supplementary material for: Psi promotes Drosophila wing growth via direct transcriptional activation of cell cycle targets and repression of growth inhibitors
Source: Development. 2023 Jan 24;150(2):dev201563. doi: 10.1242/dev.201563 (PMC10110491; doi:10.1242/dev.201563)
Supplement: Supplementary information [file develop-150-201563-s1.pdf]

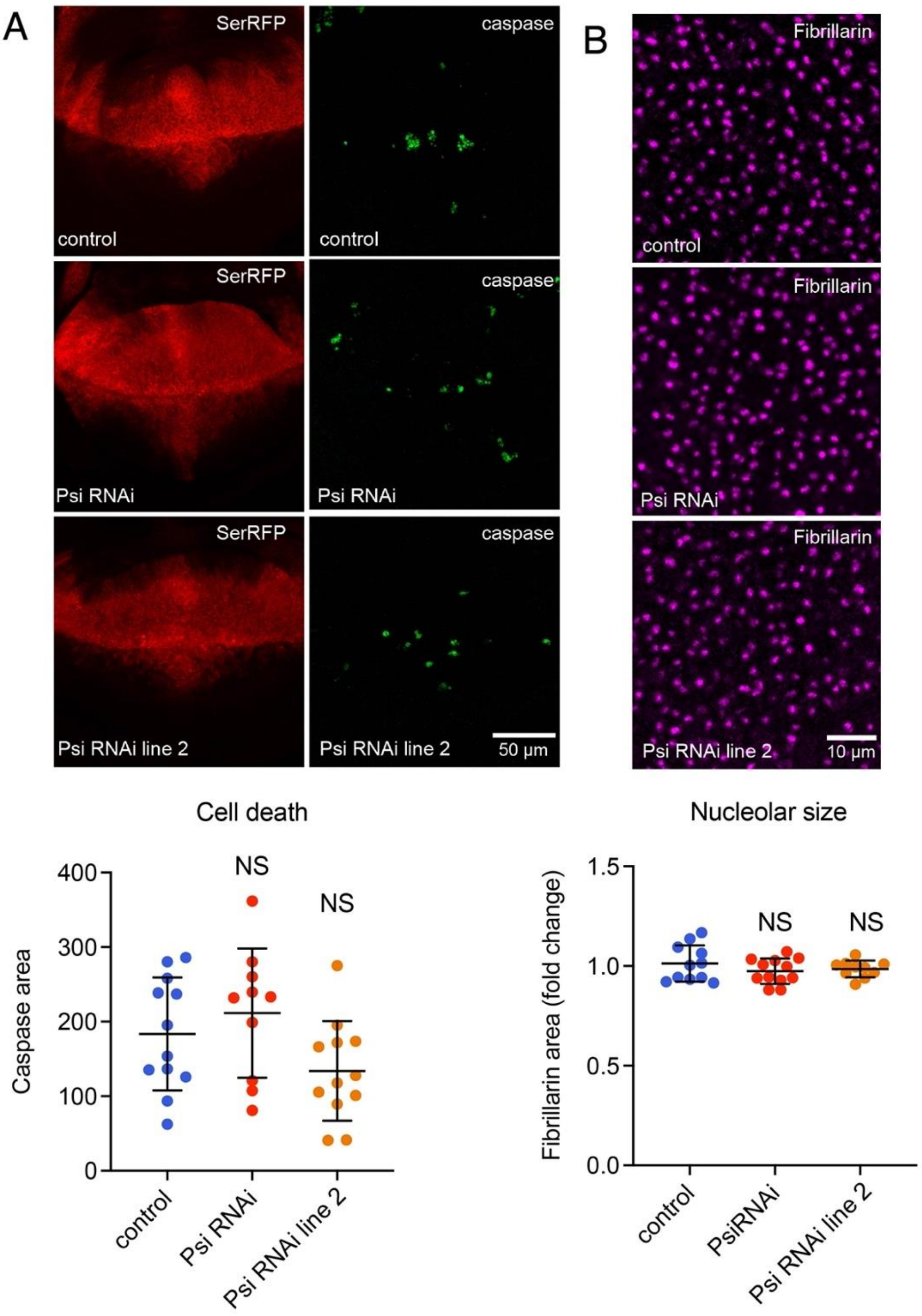

Fig. S1. Psi depletion does not affect cell death or cell growth. (A) Larval wing discs with *ser*-GAL4 expression of *Psi* RNAi or control in the dorsal compartment marked with *UAS-RFP* stained with anti-Dcp1. (B) Larval wing discs with *ser*-GAL4 expression of *Psi* RNAi or control, stained with anti-Fibrillarin. NS indicates no significance (t-test). Each data point represents a single wing disc, error bars show mean±s.d.

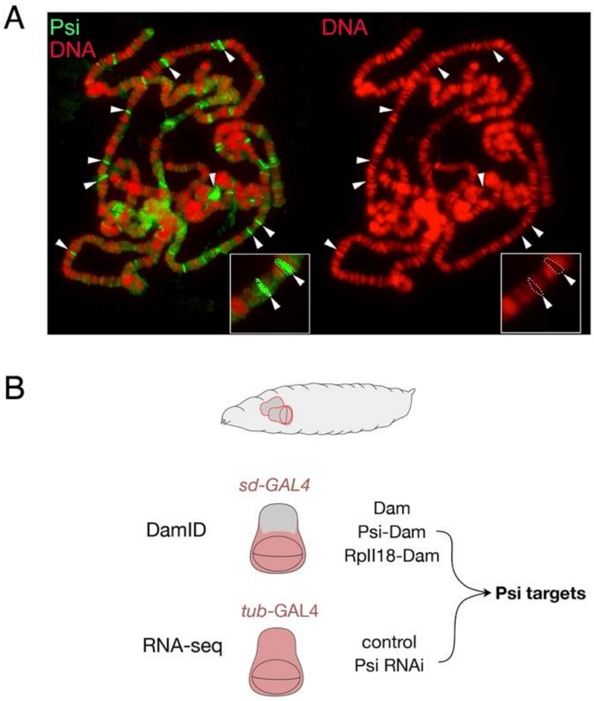

Fig. S2. Psi binds multiple genomic regions on polytene chromosomes.

(A) Salivary gland polytene chromosomes stained with anti-Psi antibody (green). White arrows indicate regions weakly stained with DAPI (red) indicative of open chromatin. (B) Strategy to identify direct genome-wide Psi targets in wing discs. RNA-seq following Psi knockdown was used to identify differentially expressed genes, and DamID for identification of direct Psi targets. DamID using RpII18 was used to monitor transcriptional state genome-wide.

Development • Supplementary information

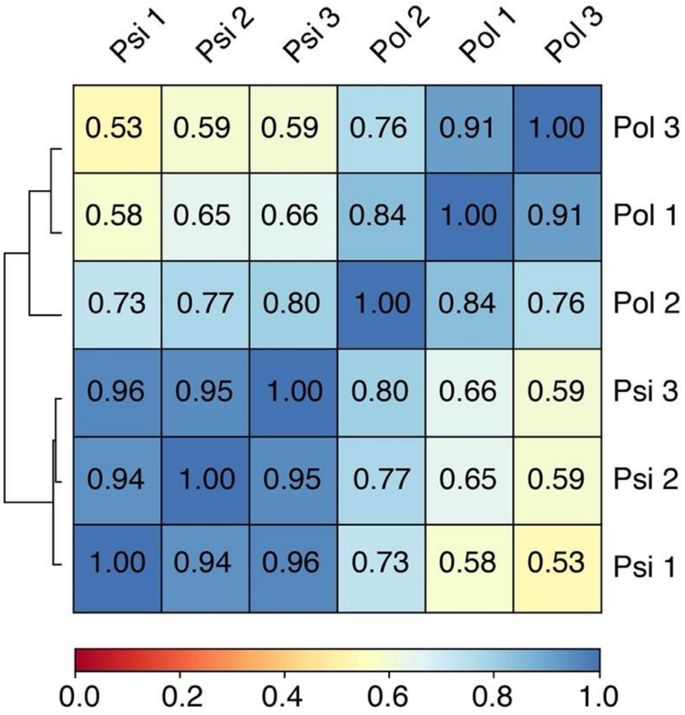

Fig. S3. DamID sample correlation. Clustered heatmap showing values for Spearman correlation of individual Psi and Pol DamID samples to verify data quality and concordance between replicate samples.

Development • Supplementary information

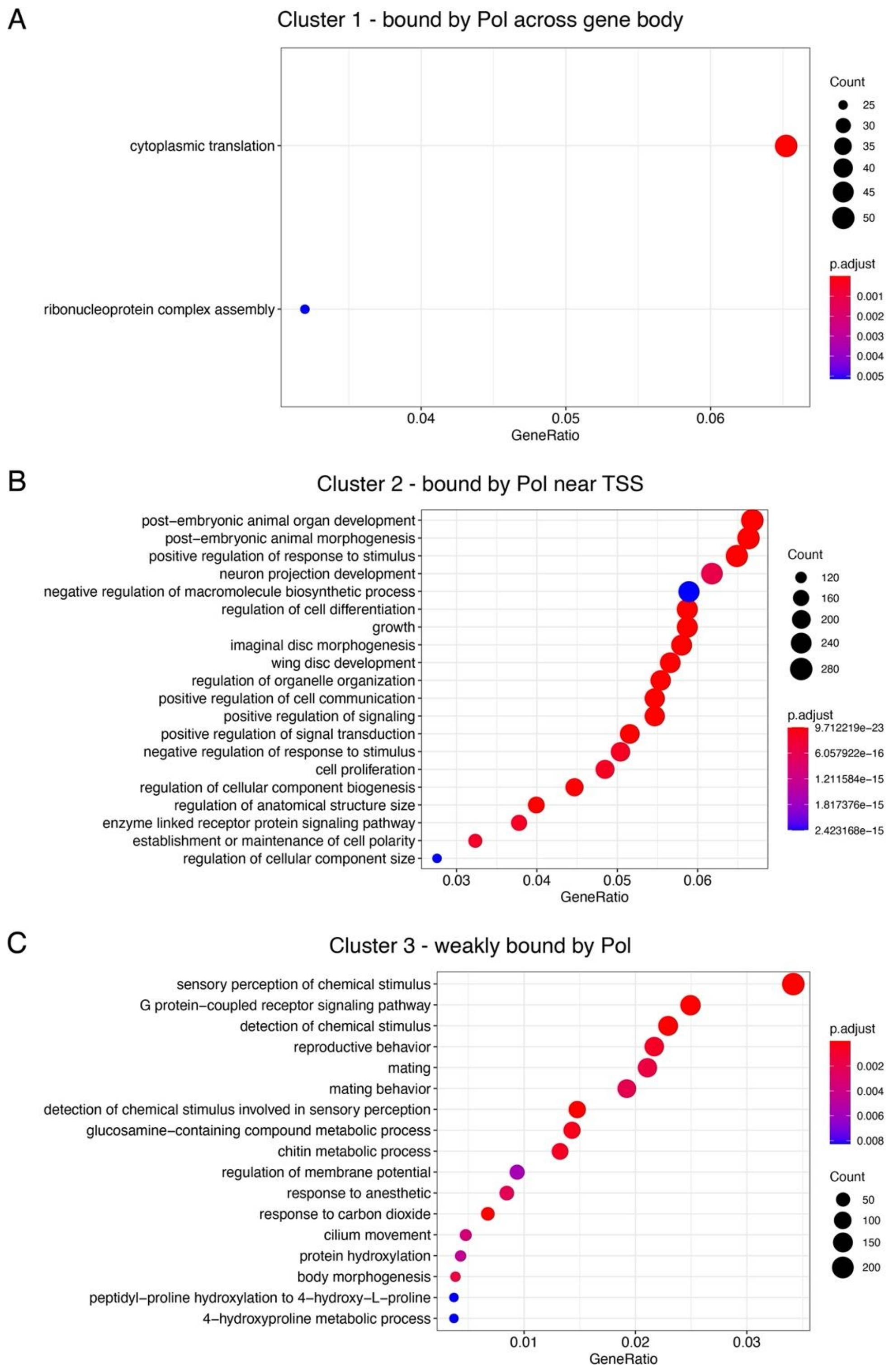

Fig. S4. Ontology of genes showing similar transcriptional activity across 3 major clusters. Analysis of gene clusters shown in Figure 2A of the main text.

Development • Supplementary information

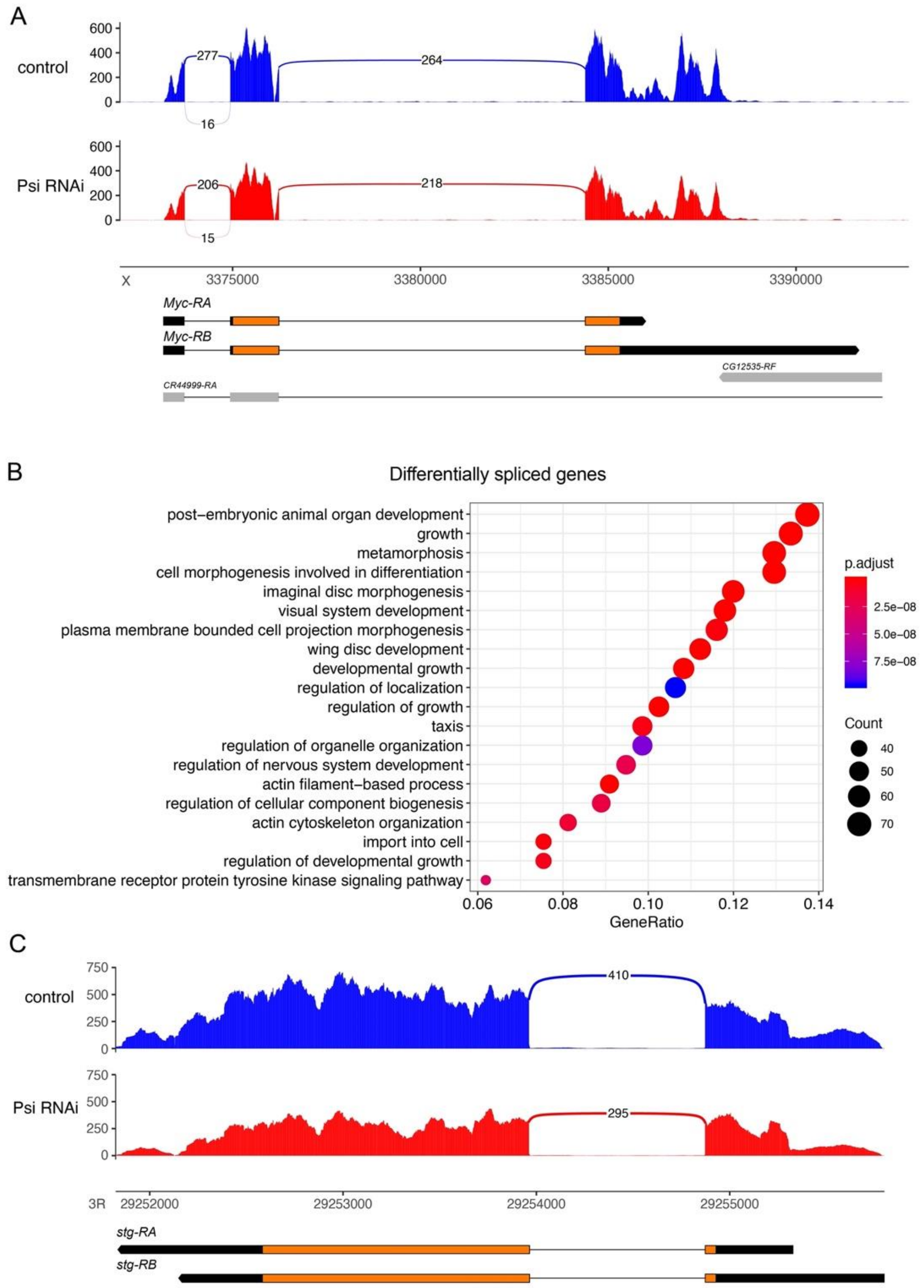

Fig. S5. Splicing analysis of *Psi* RNAi RNA-seq. (A) Sashimi plot of the *Myc* gene shown as the average of three RNA-seq replicates. (B) Gene ontology analysis of genes with differential splicing detected by rMATS. (C) Sashimi plot of the *stg* gene shown as the average of three RNA-seq replicates.

Development • Supplementary information

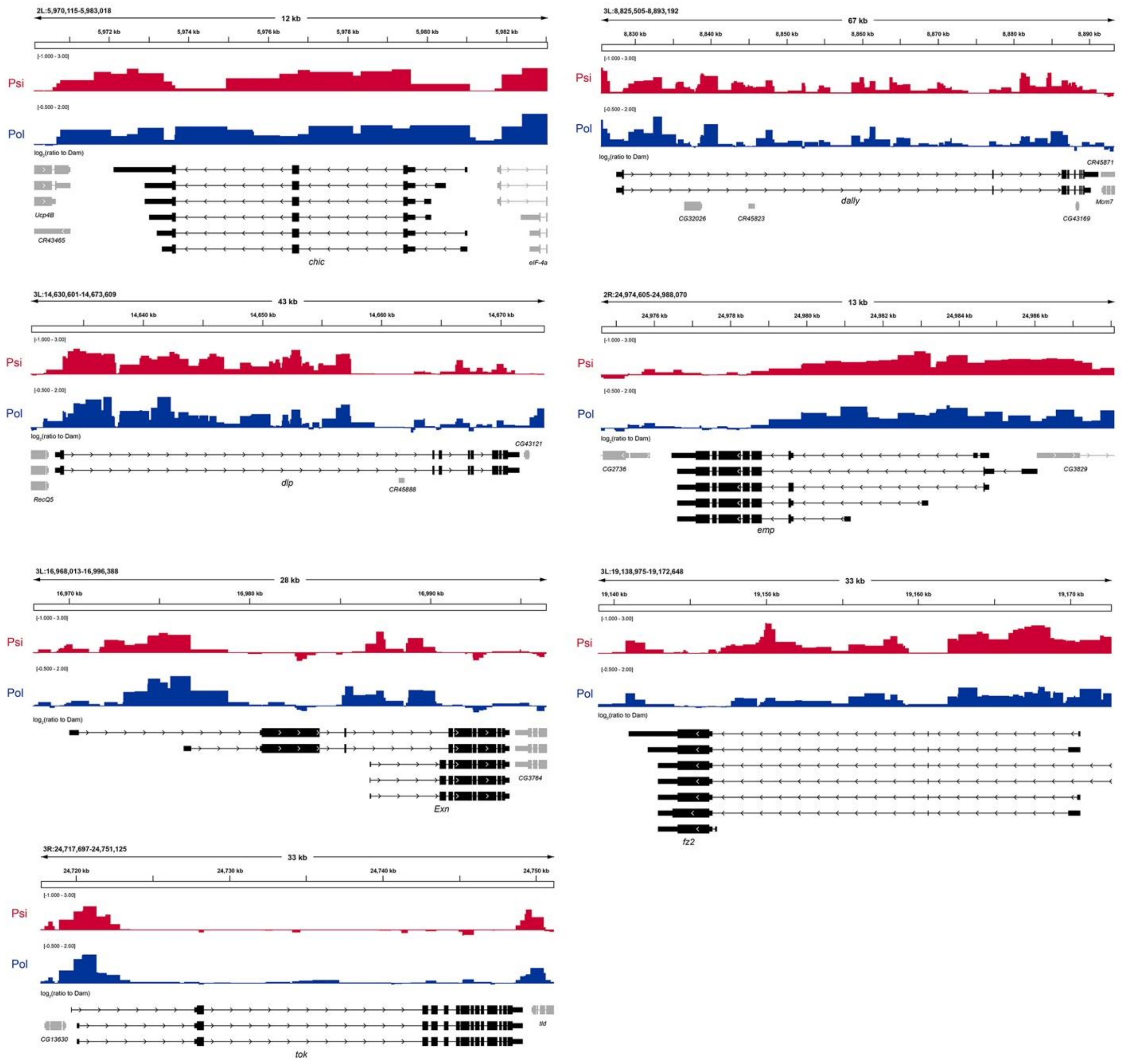

Fig. S6. Psi DamID binding profiles. Psi and RNA Pol II binding profiles across Psi's direct, repressed targets in larval wing discs that were annotated to have functions in developmental growth control ($\log_2$ of the ratio to Dam-alone control).

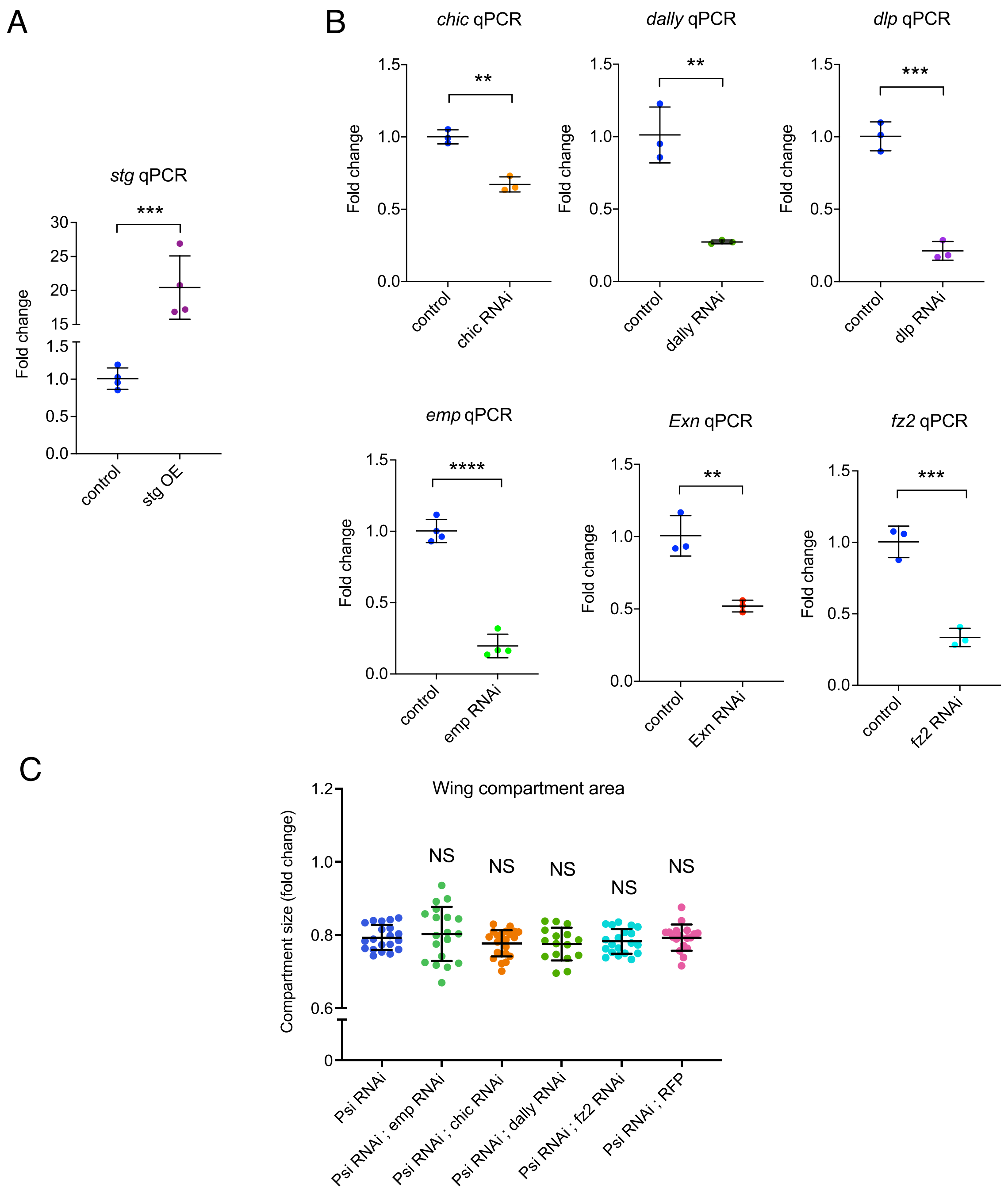

Fig. S7. Validation of transgenic lines, and Psi-dependent growth is not dependent on several negatively regulated targets. (A) qPCR of third instar larval wing discs 2 days after induction of *stg* overexpression (OE) using tsGAL80; *tub*-GAL4. (B) qPCR of third instar larval wing discs 2 days after induction of RNAi transgenes (using tsGAL80; *tub*-GAL4) for Psi targets as labelled. **p = 0.001–0.01, ***p = 0.0001–0.001, **** p< 0.0001 (t-test). (C) Quantification of the posterior compartment of the adult wing defined by the L5 vein. P-values were corrected for multiple testing using the Bonferroni method. NS=not significant. Each data point in (A-B) represents a pooled sample of 20 wing discs, data points in (C) represent single adult wings, error bars show mean±s.d.

Development • Supplementary information

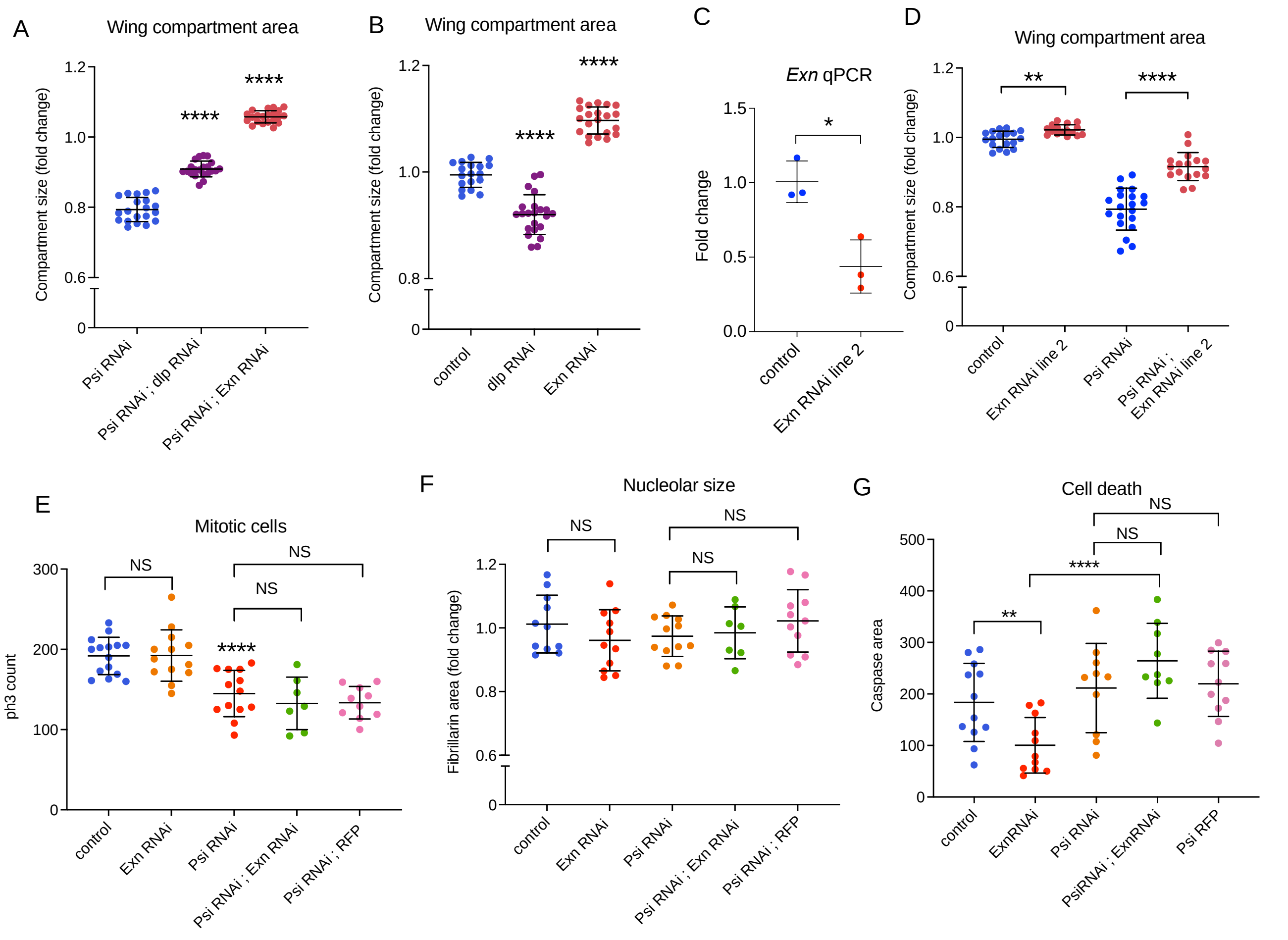

Fig. S8. Dlp KD reduces adult wing growth, while *Exn* KD rescues growth but does not modify *Psi* KD phenotypes in the larval wing. (A, B) Quantification of the posterior compartment of the adult wing defined by the L5 vein. P-values were corrected for multiple testing using the Bonferroni method. ****$p_{adj}<0.0001$. (C) qPCR of third instar larval wing discs 2 days after induction of a second non-overlapping *Exn* RNAi using tsGAL80; *tub*-GAL4. (D) Quantification of the posterior compartment of the adult wing defined by the L5 vein. P-values were corrected for multiple testing using the Bonferroni method. **$p_{adj}=0.0018$, ****$p_{adj}<0.0001$. (E) Quantification of mitotic cells in the dorsal compartment, genotypes as marked. NS = no significance, ****p<0.0001 (t-test). (F) Quantification of nucleolar area marked with anti-Fibrillarin antibody, NS = no significance. (G) Quantification of total area in the wing disc stained with anti-Dcp1 antibody. NS = no significance, **p=0.0067, ****p<0.0001 (t-test). Each data point in (A, B, D) represents single adult wings, data points in (C) represent a pooled sample of 20 wing discs, data points in (E-G) represent single wing discs, all error bars show mean±s.d.

Development • Supplementary information

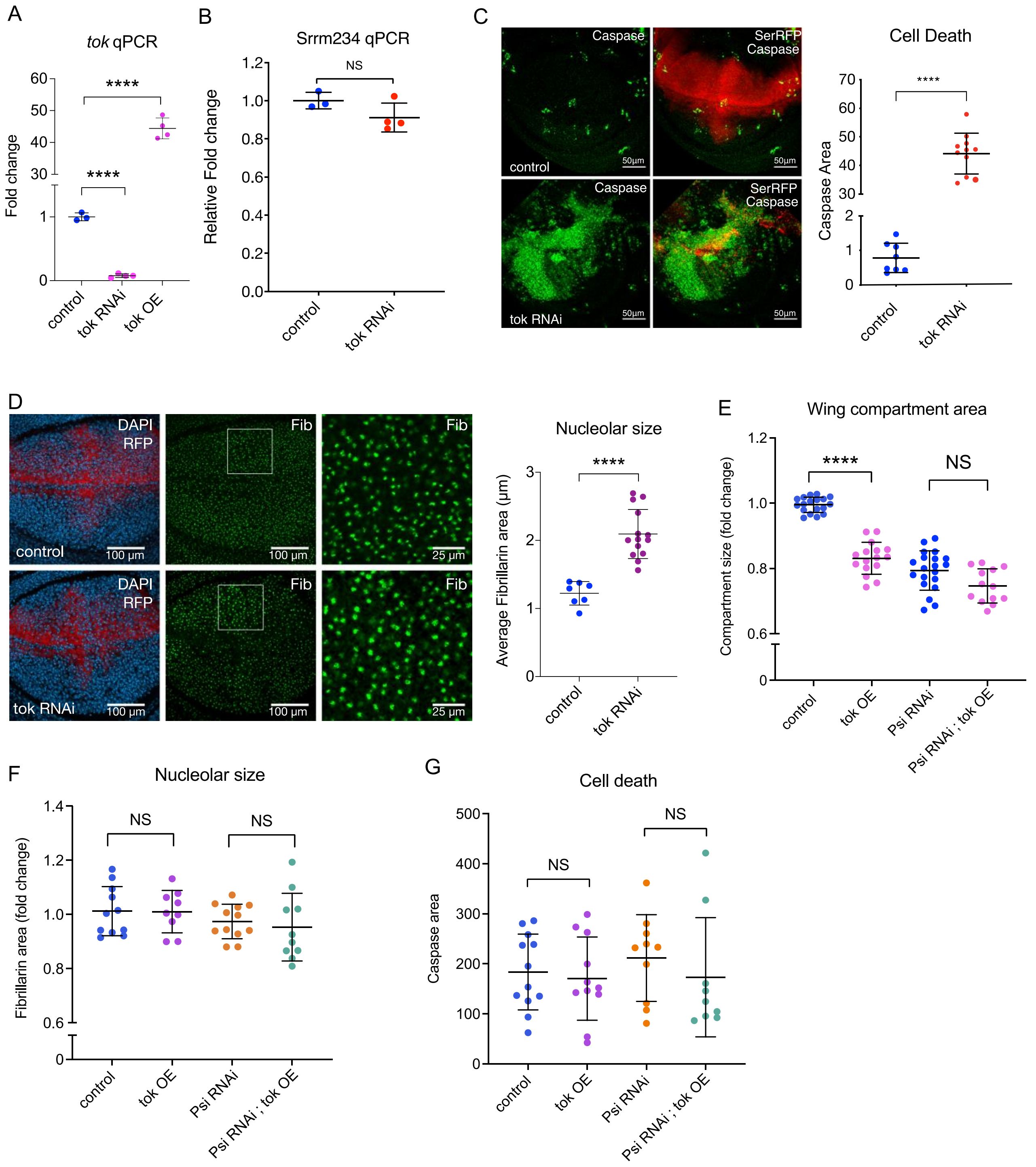

Fig. S9. *Tok* KD in the dorsal compartment significantly increases cell growth and apoptosis, while *tok* overexpression inhibits proliferation and reduces wing size. (A) qPCR of third instar larval wing discs, using tsGAL80; *tub*-GAL4 to control GAL4 activation for analysis 2 days after induction, for *tok* RNAi (TRIP line BL66320) or *tok* overexpression (OE) compared with control ****p<0.0001. (B) qPCR for predicted off target *Srrm234* in third instar larval wing discs 2 days

Development • Supplementary information

after induction of *tok* RNAi 2. (C) Larval wings stained with anti-Dcp1 for control and *tok* RNAi 2 KD (driven by *ser*-GAL4 throughout wing development). Dcp-1 area quantified as a measure of apoptosis. (D) Larval wing discs stained with anti-Fibrillarin for control and *tok* KD (24 hours after induction of KD driven by *ser*-GAL4 and marked with *UAS-RFP*). Quantification of fibrillarin area provides a measure of nucleolar size and, thus, cell growth. (E) Quantification of posterior compartment of adult wing defined by the L5 vein. NS = no significance, ****$p_{adj}<0.0001$. P-values were corrected for multiple testing using the Benjamini-Hochberg FDR method. (F) Quantification of nucleolar area (as for D). NS = no significance (t-test). (G) Quantification of anti-Dcp1 (as for C) for genotypes marked. NS = no significance (t-test). Each data point in (A,B) represents a pooled sample of 20 wing discs, data points in (C,D,F,G) represent single wing discs, data points in (E) represent single adult wings. All error bars show mean±s.d.

Table S1. Genes with significant Pol and Psi DamID binding. The profile of the normalised log_2 enrichment ratio for each GATC region calculated by the damidseq_pipeline script was analysed for significant peaks at FDR<0.01 using the find_peaks script, and peaks2genes script used to identify genes within 1 kb of the discovered peaks. Transcriptionally active genes were identified using the polii.gene.call script. For genes with significant binding of either Psi or Pol, column A contains the Flybase ID, column B contains the gene name, and column C contains the average log_2 enrichment across the significant peak corresponding to the gene.

Click here to download Table S1

Table S2. Differential gene expression analysis following Psi knockdown. Raw DESeq2 output for the analysis of differential gene expression in Psi knockdown wings compared to control, with the corresponding gene symbol in column H, EntrezID in column I and DESeq2-normalised gene counts for each sample in columns J-O.

Click here to download Table S2

Table S3. Differential splicing analysis for Psi knockdown. rMATS output of differential splicing analysis using the JCEC method (including both reads that span splicing junctions and reads on target). Individual tabs contain different analysis of various types of splicing events detected by rMATS at FDR<0.01: alternative 3'splice site (A3SS), alternative 5' splice site (A5SS), skipped exon (SE), retained intron (RI) mutually exclusive exons (MXE).

Click here to download Table S3

Table S4. Direct and differentially expressed Psi targets. Intersection of the *Psi* KD transcriptome and DNA binding profiles. Column A contains the Flybase ID, column B contains the gene name, column C contains the log_2FC value determined by the RNA-seq analysis, column D contains the average DamID enrichment for the corresponding peak, column E shows the average normalised expression across all samples, column F indicates whether differential splicing was detected (Y/N). A total of 153 genes were both bound by Psi and differentially expressed, of which 127 were not also differentially spliced.

Click here to download Table S4

Development • Supplementary information

Table S5. RpII18_gBlock sequence (lower case = Gibson assembly overlaps with pTaDaG)

ctcatctctgaagaggatctggccggcgcaCCGGCCGCCAAGCGCGTGAAGCTGGATGGCGCCGGCATGG
ATGATGCGGACTACGACAACGACGACGTTGGCGGCGATGACTTCGACGACGTCGACGA
GGACGTGGACGAGGACATTAACCAGGAGGAGGAGGCGGACAACATCGAGATCATAGCT
CCCGGTGGTGCGGGCGGAGGCGGTGTGCCCAAGTCCAAGCGCATTACCACAAAGTACA
TGACGAAATACGAGCGCGCCAGAGTTCTGGGCACACGAGCGCTTCAGATCGCCATGTGC
GCACCCATCATGGTGGAGCTGGACGGGGAAACGGACCCCCTGCAGATCGCCATGAAAG
AGCTGAAACAAAAGAAAATTCCCATCATCATCCGCCGATACCTGCCGGATCACTCCTAC
GAGGACTGGAGCATCGACGAGCTCATCATGGTGGACAACTAGgggtacctctagaggatctttgtgaag
gaa

Table S6. *Psi* ORF primers

Fwd: CTCATCTCTGAAGAGGATCTGGCCGGCGCAATGAGCGACTTCCAGCAAC

Rev: TTCCTTCACAAAGATCCTCTAGAGGTACCCTCAGTGATTGTCGTTTTTGTGC

Development • Supplementary information
